# The potential of community engagement to improve mother and child health in Ethiopia — what works and how should it be measured?

**DOI:** 10.1186/s12884-018-1974-z

**Published:** 2018-09-24

**Authors:** Peter Byass

**Affiliations:** 1 Epidemiology and Global Health, Dept. of Public Health and Clinical Medicine, Umeå University, 90,185 Umeå, Sweden; 2 Medical Research Council/Wits University Rural Public Health and Health Transitions Research Unit (Agincourt), School of Public Health, Faculty of Health Sciences, University of the Witwatersrand, Johannesburg, South Africa; 3 Institute of Applied Health Sciences, University of Aberdeen, Aberdeen, UK Scotland

Nobody in the world conceives, gestates, delivers and raises children under laboratory conditions (nor should they). But real-life variations in individual, household, environmental and health-services factors can all exert powerful influences for good and bad on maternal and child health. In combination, these factors may be synergistic, or mutually negating, in terms of overall mother and child outcomes. On top of that, there can be a number of players in any setting trying to improve mother and child health outcomes by means of various interventions, with varying degrees of coordination. Naturally any intervention investment wants to be demonstrably successful, bringing further vested interests into play in terms of attributing specific inputs to overall outcomes. The complexity of attributing specific real-life inputs to improvements in outcomes is therefore very considerable, and evaluation methods and approaches have to be found that generate robust evidence but also reflect the real-life conditions in which they must be used.

Prospective community randomised controlled trials might appear to be the optimal way of demonstrating causality between, for example, a specific health service intervention and child mortality outcomes. However, in reality the coincidental construction of a new highway linking some selected trial communities to health facilities during the period of observation, thus changing people’s access to care, might totally skew the results. The epidemiological challenge, therefore, is to find ways of linking changes in key outcomes to real-life initiatives that are intended to change those outcomes, using methods which are also reasonably practicable and cost-effective to implement as monitoring and evaluation strategies.

Ethiopia is a huge and complex country, which, at the 1990 baseline for the Millennium Development Goals (MDGs), was coming to the closing stages of a crippling civil war and faced huge challenges in many health sector outcomes, including those relating to maternal and child health. Consequently there was huge scope for improvement, and Ethiopia registered some of the most promising reductions in under-five and maternal mortality among African countries during the MDG period (1990 to 2015), with 67% and 71% declines respectively [1].

The series of papers in this Supplement all examine community engagement factors which may have contributed to recent improvements in maternal and child health in Ethiopia, even though the authors are by no means claiming that observed overall improvements are solely attributable to these factors, nor even to community engagement as a whole.

The first paper examines possible correlations between the development of Ethiopia’s Women’s Development Army (WDA), also known as the Health Development Army (a community-based strategy involving women and health extension workers and improvements in mother and child health outcomes) [2]. Unsurprisingly, from inception, WDA activities developed at different rates in different places. Thus the authors were able to use survey data from over 12,000 women in over 400 communities, correlating the density of the active WDA intervention with relevant outcomes. Higher WDA density was particularly associated with households having latrines, having visits from health extension workers, having Family Health Cards, using contraception, attending antenatal care at least four times and delivering in facilities, with rates around ten percentage points higher across these parameters for higher WDA density.

The second paper examines the effects of using Community-Based Data for Decision-Making (CBDDM) on maternal and child health care practices [3]. Before-and-after survey data from 177 communities were used to examine correlations between CBDDM strength and health care practices, in analyses that determined the effects of CBDDM. The average treatment effect of CBDDM was highest for institutional delivery (15 percentage-points out of a before-after change from 9 to 53%), though the corresponding before-after change in attending antenatal care at least four times from 28 to 52% was not associated with any statistically significant CBDDM treatment effect.

The third paper examines the effects of a Participatory Community Quality Improvement (PCQI) strategy on mother and child health care behaviour [4]. Using a similar approach to the second paper, here the authors examine the effects of PCQI over and above an emergency obstetric service intervention in certain communities. In contrast to the findings in the second paper, the average treatment effects of PCQI were highest for antenatal care (14 percentage-points for at least four antenatal visits), but also statistically significant for institutional delivery (11 percentage points).

The fourth paper examines potential effects of a specific intervention, the Family Conversation, which is a structured dialogue between a health worker, a pregnant woman and key members of the woman’s immediate family and household [5]. Statistically significant average treatment effects for Family Conversations of between 7 and 16 percentage points were observed in relation to institutional delivery, early postnatal care, clean cord care and thermal care for the newborn.

Clearly, all four papers reflect some positive associations between community engagement interventions delivered and increased levels of various mother and child health-promoting behaviours. Interestingly, the World Health Organization in its new General Programme of Work (GPW13) has made a very clear shift towards measuring health outcomes rather than outputs [6]. All the interventions described here are good examples of outputs, in that actions were demonstrably taken that might reasonably be assumed to affect outcomes – which in this case would ultimately be the effects of better health understandings and behaviours on maternal and child mortality. The difficulty therefore remains in joining the dots between outputs delivered and outcomes changed as a result. In this situation, given that other evidence suggests maternal and child mortality simultaneously declined in Ethiopia, it is very tempting to extrapolate to the conclusion that community engagement in health behaviour prevents deaths. This is probably true – but the evidence in this series of papers does not unequivocally show that to be the case.

Many other studies of maternal and child health interventions in Ethiopia have been published – too many to review systematically here. Examples include a case study of how Ethiopia reached MDG4 [7]; analyses of how disease-specific interventions may have contributed to child mortality reduction (though noting that these do not fully explain the observed mortality reduction) [8]; fairly low utilisation of maternal health services [9]; and associations between providing transport for women in labour and maternal mortality. [10] The overall picture is undoubtedly one of improvement – in terms of outputs such as services provided and community engagement with key health issues showing associations with outcomes like reductions in maternal and child mortality.

It seems clear from the papers in this Supplement that community engagement can be an important component in attempts to improve the health of mothers and children. Quite what proportion of overall health gains may be attributable to community engagement remains a tricky question, however, not least because of the lack of detailed data. In many ways the missing infrastructures in all of this are the comprehensive health information systems at the individual level which are lacking across most of Africa. Consequently, the epidemiological difficulties in linking citizens’ circumstances, health seeking behaviours, illnesses and ultimately mortality continue to create confusion in terms of which health interventions really constitute value for money in complex real-life settings. Can African countries afford not to document the lives of their citizens adequately?
